# Recent Progress in Long-Range Brillouin Optical Correlation Domain Analysis

**DOI:** 10.3390/s22166062

**Published:** 2022-08-13

**Authors:** Yahui Wang, Mingjiang Zhang

**Affiliations:** 1 Key Laboratory of Advanced Transducers and Intelligent Control System, Ministry of Education and Shanxi Province, Taiyuan University of Technology, Taiyuan 030024, China; 2 College of Physics, Taiyuan University of Technology, Taiyuan 030024, China

**Keywords:** fiber optics sensors, stimulated Brillouin scattering, Brillouin optical correlation domain analysis, long range, chaotic laser

## Abstract

Distributed optical fiber sensing technology has been widely applied in the areas of infrastructure health monitoring, national defense security, etc. The long-range high-spatial-resolution Brillouin optical correlation domain analysis (BOCDA) has extensive development and application prospects. In this paper, long-range BOCDAs are introduced and summarized. Several creative methods underpinning measurement range enlargement, including the interval enhancement of the adjacent correlation peak (CP), improvements in the signal-to-noise ratio, and the concurrent interrogation of multiple CPs, are discussed and experimentally verified, respectively. The main drawbacks in the present BOCDA schemes and avenues for future research and development have also been prospected.

## 1. Introduction

Over recent decades, Brillouin optical correlation domain analysis (BOCDA) technology based on stimulated Brillouin scattering (SBS) has found various major applications for temperature, strain, and variation sensing, such as structural health monitoring, aerospace, and military defense [1,2,3,4]. Compared to the pulse-based time-domain technology, BOCDA can circumvent the limitations of the phonon lifetime (on the order of 5–10 ns) on pulse width to achieve a centimeter- or millimeter-scale spatial resolution (SR) [5,6,7]. Benefiting from the high SR, BOCDA has great potential in distinguishing small-size events and precise micro-scale measurements, based on a relatively long sensing distance [8,9].

In the BOCDA scheme, the SBS acoustic field is stimulated at a particular fiber location, where the optical pump and probe waves are highly correlated. Concretely, the joint modulated pump and probe waves are counter-propagated and the SBS process is closely determined by the temporal cross-correlation function between the two waves at a point of interest. The SBS interaction is largely confined to discrete positions, referred to as correlation peaks (CPs), and that of other positions is significantly unexcited. Consequently, the intensity of acoustic wave is restricted to the CP width, whose spatial extent is defined as the theoretical SR [10]. Remarkably, the Brillouin interaction at CP positions is constant and stable, so that there is no limitation of the excitation time, i.e., the SR can break through to a centimeter or millimeter scale.

In 1999 and 2000, the first realization of BOCDA was proposed based on a frequency modulation (FM) source using a sinusoidal wave, where a 40 cm resolution is demonstrated, and the obstacles for the conventional pulse-based techniques are overcome [11,12]. Moreover, several enhanced schemes have been proposed to achieve a higher resolution, such as the three-electrode laser with a large push–pull-type current [13], intensity modulation [14,15,16], convexity extraction algorithm [17], etc. [18,19]. However, the multiple CPs generated by periodic signals can simultaneously exist along with the fiber under test (FUT) and then will give rise to the ambiguous measurement of the sensing distance, resulting in a trade-off problem between the measurement range (MR) and SR in principle [12]. To enlarge the MR, three strategies have been proposed: extending the interval of adjacent CPs, concurrently interrogating multiple CPs, or improving the measurement SNR.

For extending the CP interval, the temporal gating [20,21], pump–probe switch [22], and frequency chirp magnification [23] have been applied in the sine-FM BOCDA. Further, a novel BOCDA scheme based on the phase-modulated (PM) source has been proposed and demonstrated since 2012, where the pump and probe waves are jointly phase-coded by pseudo-random bit sequence (PRBS) [24]. The SR is closely related to the duration of a single bit and the periodic interval is determined by the length of the sequence, which may be arbitrarily long. Consequently, PRBS-PM BOCDA can greatly remove the constraint between the MR and SR, ignoring the limitation induced by the code length, the pseudo-random property, and off-peak interactions. Therefore, the broadband source, such as amplified spontaneous emission (ASE) [25] and chaotic laser [26], has been introduced into the BOCDA system to generate a sole CP over an arbitrarily long FUT due to their noise-like characteristics. The broadband source-based BOCDA can eliminate the periodicity of CPs, thus avoiding the gain crosstalk and expanding the sensing distance, where the inferior signal-to-noise ratio (SNR) limits the further improvement of MR [25,26]. 

For interrogating multiple CPs concurrently, the bidirectional detection, frequency division multiplexing, and time division multiplexing technologies have been applied, not only in the sine-FM schemes but also in the PM BOCDA [27,28,29,30]. Meanwhile, several SNR-enhanced methods, such as differential measurement [31], Golomb coding [32], time-delay signature (TDS) suppression [33,34], and distributed Raman amplification (DBA) [35], have been proposed and demonstrated to extend the MR, which can reach 52 km [35]. 

This paper reviews the recent progress in long-range BOCDA. In Section 2 of this paper, the principle of BOCDA is introduced and several extensions from it are presented from three aspects. In Section 3, varieties of inspired methods for enlarging MR are addressed based on the different modulation types. This review aims to give a summarized perspective on the development process and the state of the art of these protocols. A conclusion and prognosis are given in Section 4. 

## 2. Operating Principle

### 2.1. SBS Interactions

The SBS is a typical amplification process resulting from the electrostriction effect [36]. The pump and probe waves counterpropagating in the FUT interfere and excite the acoustic wave, and the refractive index grating is generated by coupling the three waves. As a result, optical power is transferred from the pump to the probe, and the intensity of the probe wave is amplified. Remarkably, the acoustic wave is forward-propagated and a Doppler shift is added to its backscattered Stokes wave, where the frequency offset ($\nu_{B}$) is known as the Brillouin frequency shift (BFS) is and given by:

(1) $$\nu_{B}=\frac{2nV_{a}}{\lambda }$$

where *n* is the refractive index of FUT, $V_{a}$ is the velocity of the acoustic wave, and λ is the central wavelength of the injection light. For the λ of 1550 nm, the $\nu_{B}$ of a normal single-mode fiber (SMF) is around 10~11 GHz.

The above SBS process can be expressed by the coupled three-wave equations:

(2) $$\frac{\partial A_{P}}{\partial z}+\frac{1}{V_{g}}\frac{\partial A_{P}}{\partial t}=\frac{i\pi \gamma_{e}}{2n\lambda \rho_{0}}\rho A_{S}$$

(3) $$-\frac{\partial A_{S}}{\partial z}+\frac{1}{V_{g}}\frac{\partial A_{S}}{\partial t}=\frac{i\pi \gamma_{e}}{2n\lambda \rho_{0}}\rho^{*}A_{P}$$

(4) $$\frac{\partial \rho }{\partial t}+(\frac{\Gamma_{B}}{2}-i\Gamma ’)\rho =\frac{\pi n\varepsilon_{0}\gamma_{e}}{4\lambda V_{a}}A_{P}A_{S}^{*}$$

where $A_{P}$, $A_{S}$, and ρ are defined as the slowly varying complex amplitude of the pump wave, the Stokes wave (i.e., the probe wave), and the acoustic wave, respectively. $V_{g}$ is the group velocity of light in fiber, $\varepsilon_{0}$ is the dielectric constant, $\gamma_{e}$ is the electrostriction coefficient, and $\rho_{0}$ is the mean density of the fiber. The above parameters are directly related to the fiber materials. $\Gamma_{B}$ is the linewidth of the Brillouin gain spectrum (BGS) and Γ’ is the relative detuning frequency between the pump and the Stokes waves. The SBS amplification can reach the maximum only after meeting the phase-match state, i.e., Γ’=0. Consequently, in Brillouin scattering-based sensors, the optimized SBS interaction can be stimulated by scanning the pump and the probe wave frequency difference, detected by measuring the BGS, which can be expressed as:

(5) $$g=g_{0}\frac{{\left(\Gamma_{B}/2\right)}^{2}}{{\left(\Omega_{B}-\Omega \right)}^{2}+{\left(\Gamma_{B}/2\right)}^{2}}$$

Remarkably, the SBS interaction automatically matches the phase condition, meaning that the Brillouin gain, g,reaches the peak value at $\Omega_{B}=\Omega$ ($\Omega_{B}=2\pi \nu_{B}$ is the angular BFS, and Ω is the angular frequency difference between the pump and the Stokes waves). The full width at half maximum (FWHM), $\nu_{B}$, of the intrinsic BGS is around 30 MHz, meaning that $\Gamma_{B}=2\pi \cdot 30\;\mathrm{MHz}$ [37]. The local BFS is obtained by fitting the BGS and picking the peak value, which is linear with the variations of temperature or strain, so the distributed measurement can be achieved by obtaining and demodulating the BGSs.

In fact, the measured BGS is a convolution of the natural one with the beat spectrum, which is equivalent to the pulse spectrum in time-domain sensors and the pump-probe beat spectrum in correlation domain sensors [12]. The influence of the pulse width on the BGS has been investigated, in light of the fact that the spectrum is significantly broadened and the intensity is drastically reduced when the pulse width decreases. The FWHM of the 10 ns pulse-based BGS has been widened to around 100 MHz, and that of 5 ns can reach approximately 200 MHz. As analyzed by the formula of BFS accuracy, the measurement accuracy will be seriously deteriorated due to the low SNR and wide spectrum [5]. Therefore, a trade-off between the pulse width and measurement accuracy directly restricts the SR of pulse-based sensors, which is on the order of several meters with the MR of tens of kilometers. 

Moreover, many effective techniques have been developed for centimeter-level SR, such as the dark pulse [38], the pre-pump pulse [39], and the differential pulse–width pair [40]; however, the sensing distance is limited to thousands of meters due to the low SNR and non-local system response [41]. For example, in the dark pulse scheme, a self-actuated SBS stimulated by the high pump power in long FUT would severely deteriorate the SNR and the MR is merely 80 m. The non-local gain response of the micro-scale BFS section will continuously decay with increasing MR. The trade-off problem between high SR and long MR is extremely challenging to the SR-enhanced BOTDA schemes. Interestingly, the unique merit of BOCDA in achieving millimeter-level SR can be paid more attention due to the implementation of long-range high-resolution sensing systems.

### 2.2. Basic Sine-FM BOCDA

The BOCDA is a novel Brillouin sensor based on SBS amplification, which can accurately achieve a high-resolution temperature or strain identification via a micro-scale acoustic field stimulated by the highly correlated laser source. 

**Figure 1:**
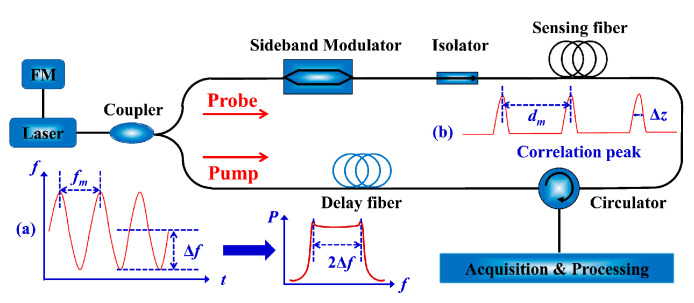
The experimental setup of the basic sine-FM BOCDA. (**a**) Map of the optical frequency distribution and the optical spectrum, (**b**) distribution of the periodic CPs.

In 2000, the basic BOCDA configuration enabled by the frequency-modulated pump and probe waves was systematically demonstrated and analyzed [12]. The experimental setup is illustrated in Figure 1. A single laser diode (LD) is typically used as the source and direct current (DC) modulated by a sinusoidal wave. The FM-modulated wave is split into pump and probe paths. The light in the probe path is modulated in the sideband suppress-carrier format using an electro-optic modulator (EOM), driven by a microwave generator (MWG) to match the BFS of the fiber, and the light in the pump is transmitted through a delay fiber. Then, the pump and probe waves are launched into two opposite ends of the FUT via an isolator or optical circulator (OC), respectively. The data acquisition and processing model is placed at the output of the OC to collect the gain signal. As shown in the inset view (a), the optical frequency is modulated into sinusoidal distribution, where the modulation frequency $f_{m}$ and the modulation amplitude Δf are determined by the initial bandwidth of the sine wave and the DC modulation depth, respectively. The FM-modulated laser performs the characteristics of saddle-shaped broadband optical spectrum with a linewidth of 2Δf. Based on the synthesis of the optical coherence function (SOCF) [42,43], the highly correlated probe and pump waves will generate a series of periodic CPs at the specific positions, as illustrated in the inset view (b). The CP width, Δz, and the interval of adjacent CPs, $d_{m}$, can be expressed as:

(6) $$\Delta z=\frac{V_{g}\Delta \nu_{B}}{2\pi f_{m}\Delta f}$$

(7) $$d_{m}=\frac{V_{g}}{2f_{m}}$$

**Figure 2:**
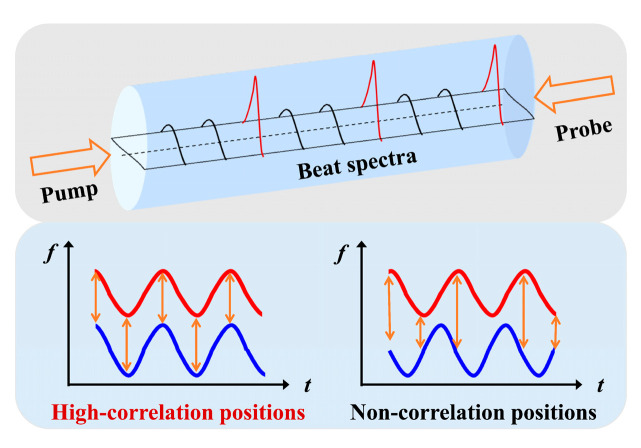
Schematic diagram of operating principles in sine-FM BOCDA.

Concretely, as illustrated in Figure 2, the jointly modulated pump and probe waves are launched into FUT from the two opposites, respectively. The frequency difference, i.e., the beat frequency, between the pump and probe waves can be written as [15]:

(8) $$f_{b}(t)=\Delta \nu +\Delta z\cdot \left\{\sin(2\pi f_{m}t)-\sin(2\pi f_{m}(t-2\Delta x/V_{g}))\right\}$$

where Δx is the propagation length difference between the pump and probe beams. When the optical path difference between the two beams is an integral multiple of $d_{m}$, the probe and the pump waves maintain a constant frequency difference, where a stable interference beat effect will appear at these operating positions (i.e., CP positions). The intensity distribution of beat spectrum is presented as a δ function, and a sharp SBS gain can be obtained in any independent CP. When the operating positions of the two beams deviate from the CP, the frequency difference rapidly changes and the beat spectrum is gradually broadened. Therefore, the stable SBS acoustic field cannot be effectively excited and the weak noise floor can be continuously motivated along the FUT. 

Obviously, the SR, Δz, of sine-FM BOCDA is directly related to the $f_{m}$ and Δf; as such, two modulation parameters are larger and the SR is higher. Unlike the spatiotemporal mapping of the pulse signal in time-domain technology, the acoustic field can be adequately excited by the narrow CP with time continuation and space independence properties, so that the SR can circumvent the phonon lifetime limitation from reaching the millimeter scale. However, the multiple discrete CPs excited at the same time along the FUT can result in a significant gain ambiguity, which leads to the theoretical MR equivalent to $d_{m}$. In the proof-of-concept experiment, a 40 cm SR is preliminarily obtained over a 30 m MR and the fully distributed measurement can be achieved by adjusting the $f_{m}$ to localize the CP positions [12]. Apparently, the predicament that the modulation frequency and amplitude cannot be simultaneously increased results in a significant limitation in the sensing range, although the SR can be promoted to 1.6 mm [13]. 

### 2.3. Basic PRBS-PM BOCDA

**Figure 3:**
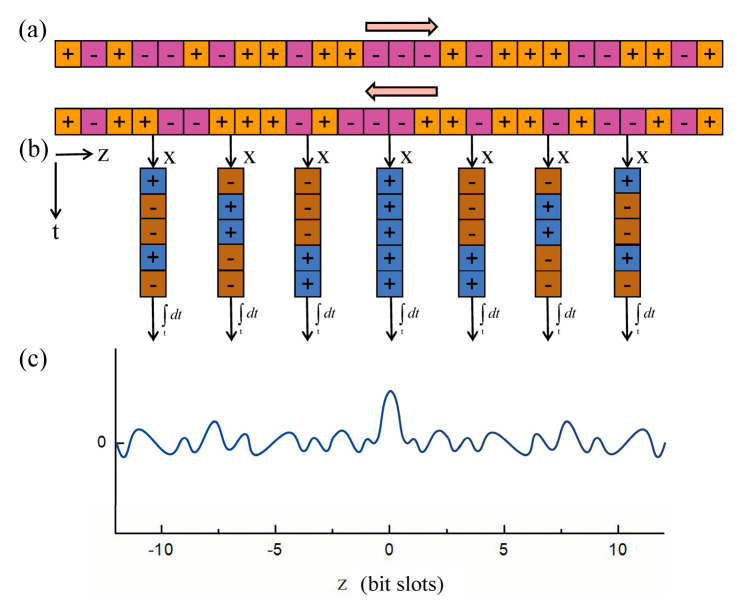
Schematic diagram of operating principle of PRBS-PM BOCDA [21]. (**a**) The PRBS sequence. (**b**) The instantaneous driving force for the generation of the SBS acoustic field. (**c**) The magnitude of the resulting acoustic field.

For extending the interval of adjacent CPs, A. Zadok and co-authors proposed the PRBS-PM BOCDA [24]. The sensing principle is illustrated in Figure 3. Similar to the sine-FM waves, both pump and probe waves are jointly phase-modulated by a common PRBS, whose duration is much shorter than the phonon lifetime. The modulation phase within each symbol assumes a value of either 0 or π, with equal probabilities. As Equation (8) shows, where $\Gamma =\Gamma_{B}/2-i\Gamma ’$, $\theta (z)=\Delta x/V_{g}$ is a position-dependent time lag and *u(t)* denotes a definite modulation function of the optical source. The instantaneous driving force for the SBS acoustic field generation is proportional to the product of the pump wave envelope and the complex conjugate of the probe wave.

(9) $$\bar{Q(z,t)}=j\gamma_{e}\int_{0}^{t}\exp\left[-\Gamma (t-t^{\prime })\right]\;\bar{u\left(t^{\prime }-\frac{z}{V_{g}}\right)u^{*}\left[t^{\prime }-\frac{z}{V_{g}}+\theta (z)\right]}dt^{\prime }$$

The pump and probe waves are highly correlated in the vicinity of the FUT center, meaning that their phase difference is constant and the driving force keeps a steady non-zero value. Consequently, the acoustic field is allowed to build up to its steady-state value. In all other locations, the driving force rapidly fluctuates due to the randomly alternating phase difference. The magnitude of acoustic field magnitude thus averages out to zero for all non-correlated times; hence, the SBS interaction outside the CP is largely inhibited [24]. The profile of the acoustic field that is generated in the PRBS-PM system is equivalent to the sine-FM scheme so that the width and interval of the CPs can be expressed as:

(10) $$\Delta z=\frac{1}{2}V_{g}T_{b}$$

(11) $$d_{m}=\frac{1}{2}MV_{g}T_{b}=M\cdot \Delta z$$

where the $T_{b}$ is the symbol duration (i.e., the reciprocal of bit rate) of PRBS and *M* is the length of that. Since the sequences of 2^31^-1 bits long can be readily generated, the MR can be largely extended and, theoretically speaking, can be arbitrarily long. The localization of CP is achieved by scanning the bit duration.

**Figure 4:**
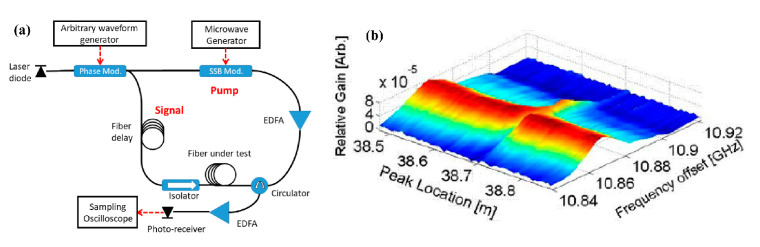
(**a**) Schematic diagram of experimental setup of PRBS-PM BOCDA. (**b**) Brillouin gain map in the vicinity of the 1 cm heated section of the 40 m FUT [24].

An experimental setup of PRBS-PM BOCDA is shown in Figure 4a. A single LD is employed as the source and an electro-optic phase modulator (EOPM), driven by a PRBS generator, is placed after that to repeatedly generate the highly correlated laser, which is separated into pump and probe waves. Slightly different from the basic sine-FM scheme, a single-sideband (SSB) modulator is shifted to the pump branch, which is also driven by a MWG, and the delay fiber is moved to the probe branch. The pump, amplified by an erbium-doped fiber amplifier (EDFA), and probe waves are lunched into the FUT though the OC and isolator, respectively. The signal wave, amplified by the Brillouin interaction, is acquired by a photo receiver and sampled by an oscilloscope.

Figure 4b presents the corresponding experimental result, when the generator clock rate $1/T_{b}$ is in the range of 8–12 GHz, corresponding to Δz between 0.9 cm and 1.3 cm, and the code length is M = 2^15^ − 1. The SBS gain is shown as a function of the position and the BFS. The MR is 40 m and the SR is 1 cm, where a 1 cm hot spot is properly identified. However, with an increase in the FUT length, the variance of the acoustic wave magnitude at off-peak locations, as shown in Figure 3c, will accumulate over the entire length and lead to a significant fluctuation in the output power. This non-negligible phenomenon induces a dominant noise mechanism and the Brillouin gain within the short correlation peak may become submerged, resulting in the MR still being greatly limited [24]. 

### 2.4. Basic Broadband Source-Based BOCDA

To overcome the limitation of periodic CPs, an aperiodic peak approach can be firstly taken by using the polarized ASE of an EDFA as the source of the Brillouin pump and probe waves [25]. Different from degrading the initial coherence of the laser through the FM or the PM, the light of the ASE source is highly incoherent to begin with, where the modulation function *u*(*t*) is no longer deterministic. Both the amplitude and phase randomly fluctuate and then the SBS interaction is confined to a single CP due to the δ-like cross-correlation function. The SR theoretically equals to the coherence length of the ASE source and might be as short as tens of microns by tuning the length to hundreds of GHz. ASE-based BOCDA is experimentally realized with a MR of 50 mm and a SR of 4 mm, which is practically restricted by poor SNR due to the low spectral density, resulting in a weak intensity fluctuation in the time trace and strong amplitude noise in the autocorrelation function. In addition, the power of the gain signal is relatively feeble compared to that of the inherently stochastic fluctuations [44].

**Figure 5:**
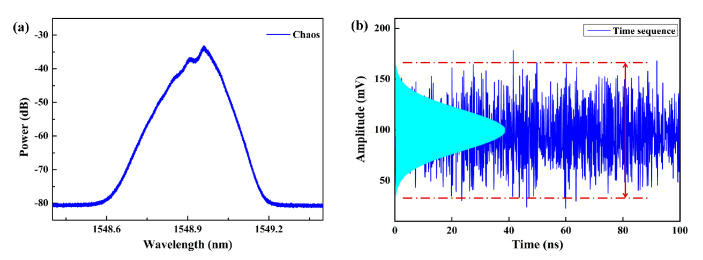
(**a**) Optical spectrum and (**b**) time series of the chaotic laser.

Further, another competitive protocol of BOCDA that shares some of the properties of the ASE-based approach is proposed and experimentally demonstrated by M. Zhang et al. (author’s team) [26]. In this approach, a chaotic laser, which is generated by a semiconductor laser with external perturbations, is employed as the laser source [45,46,47]. Similar to the ASE light, chaotic laser features as the wideband spectrum in phase and randomness in amplitude,, which may be referred to as a stationary Gaussian random process, as depicted in Figure 5 [48]. The noise-like waveform of several GHz-scale linewidths and the δ-like autocorrelation function, *u*(*t*), which is also stochastic, can be used with the Brillouin pump and probe waves, and then the driving force of the SBS acoustic field is generated within the sole CP. When the optical path of the two beams is equal, implying that a constant phase and amplitude difference exist at a specific position, a stable interference beat effect will appear and the sole CP will be generated. When the operating positions deviate from the CP, the beat spectra are gradually broadened and the stable SBS acoustic field cannot be effectively excited. Therefore, the SR of the chaos-based protocol is also determined by the coherence length, which is equivalent to the FWHM of the central CP. The MR can be theoretically extended to arbitrarily long, thus benefiting from the unambiguous gain signal. 

**Figure 6:**
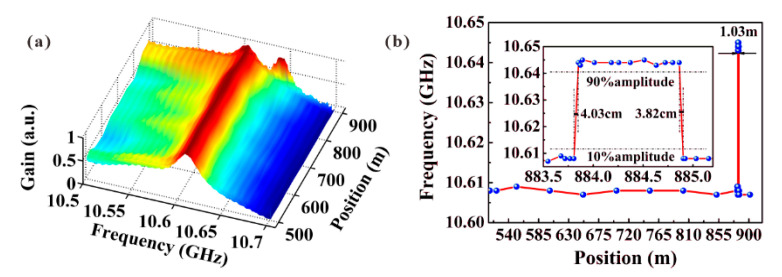
(**a**) Distribution map of BGSs along the FUT. (**b**) BFS distribution along the FUT [26].

The experimental setup used in basic chaos-based BOCDA is illustrated in [23]. Similar to the other schemes, the chaotic laser is split into pump and probe branches. The probe wave is modulated in a double-sideband suppress-carrier format and transmitted through a programmable optical delay generator (PODG), an EDFA, and a polarization scrambler (PS). The pump wave is amplified by an EDFA and then launched into the FUT using an OC. Remarkably, the single CP is localized and scanned by the PODG, whose accuracy of 0.001 mm will not deteriorate the SR [49]. The three-dimensional BGSs along the FUT are shown in Figure 6a and a heated fiber of 1 m can be clearly distinguished. The SR is estimated by the mean value of the equivalent length of rising and fall time; therefore, the SR can approximately approach 4 cm along the 906 m FUT [26].

Compared to the sine-FM or PM BOCDAs, the ASE- and chaos-based schemes are adopted as the inherently high-incoherence broadband source in the sensing signal. First, the modulation bandwidth limitation, using either DC or PM methods, can be largely solved to simply achieve a millimeter-scale SR. A second advantage is that the single CP along an entire FUT can circumvent the trade-off between the high SR and large MR in principle. In addition, chaos-based BOCDA, performing a superior SNR and sensing index, represents a potential protocol in long-range high-resolution techniques. 

## 3. Measurement Range Enlargement

### 3.1. Improvement in Measurement SNR

In the BOCDA system, Brillouin interaction is occurred by two counter-propagating high-correlated waves and detected by sampling the power variations of frequency-scanning probe light at the end of the FUT. Consequently, the measured Brillouin signal is the sum of all the stimulated gains along the entire fiber. Although the local BGS is sharp and Lorenz-shaped at the CP positions, a series of weaker BGSs can be constantly excited and accumulated to compose a strong noise background, which may submerge the central gain signal and severely restrict the MR. Nowadays, numerous impressive techniques have been introduced to suppress the noise structure and improve SNR measurements. 

#### 3.1.1. Sine-FM BOCDA

**Figure 7:**
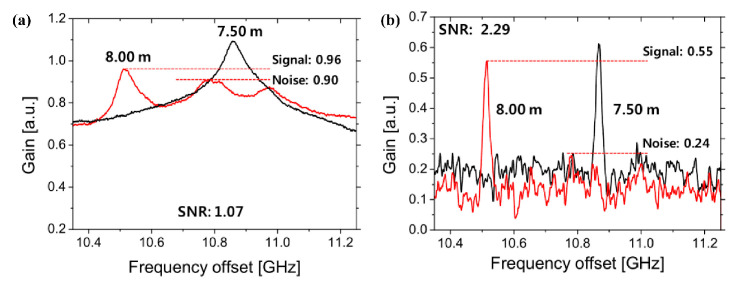
The measured BGS (**a**) before and (**b**) after differential measurement with SMF (black) and DSF (red) sections [28].

In sine-FM BOCDA schemes, the distribution gain signal coincides with that of the beat spectra. A sharp BGS generated at the CP position reflects the intrinsic local gain. On the contrary, as the position is shifted from the CP, the local BGS is broadened and spread out. The BGSs at different fiber sections of single-mode fiber (SMF) and dispersion-shifted fiber (DSF) are experimentally measured [31]. As shown in Figure 7a, in the ordinary lock-in detection scheme, with the shifting of the CP position (SMF to DSF), the gain peak gradually moves to the new BFS. However, the trapezoid-shaped noise structure, accumulated from the non-correlation positions, severely limits the SNR of BGS. With an increase in the sensing distance, the intensity of noise floor may continuously ascend and the gain peak may become completely submerged.

To suppress the noise structure, a differential measurement scheme is proposed and experimentally demonstrated [31]. A frequency of PM that is much smaller than Δf is additionally applied to the pump branch for carrier suppression and spectral broadening, the local BGS is spread out at the CP position, and the noise floor is practically measured. Remarkably, the broadening effect with the PM is the largest at the CP and becomes negligible at which the beat spectrum is already broad. As a result, the pure Brillouin gain signal may be obtained by analyzing the difference between the noise structure and the original BGS. The measured BGSs under differential measurements are illustrated in Figure 7b. It is noticeable that the noise background is largely suppressed and the gain peak is dominated although the central BFS is shifted, where the SNR of BGS is improved to 2.29 dB. The spectral width of pure BGS is much smaller than that of the ordinary one due to the almost complete suppression of the noise structure. Additionally, the effects of differential measurement are analyzed, and a six-fold improvement in SR, which is enhanced three-fold for a long-range system, is achieved by optimizing the parameters of the PM [50].

#### 3.1.2. PM-BOCDA

Similarly, a high-rate PRBS is used in PM BOCDA measurements so that the amplitude variance of the acoustic wave at off-peak positions (i.e., non-correlation positions) for a certain frequency can also fluctuate and become instable. Off-peak SBS interactions accumulate over the entire FUT and then lead to undesirable fluctuations in the power of the output signal. This structure gradually becomes the dominant noise mechanism in PM BOCDA and might conceal the Brillouin gain within the short CP. The optical signal-to-noise ratio (OSNR) is defined as the ratio between the coupling power at CP and the variance in the output power, which can be estimated by a simple relation [10]:

(12) $$\mathrm{OSNR}\approx \frac{2V_{g}\tau }{L}$$

**Figure 8:**
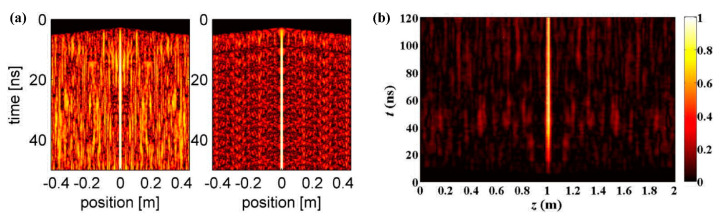
The spatio-temporal distribution of the acoustic field. (**a**) PRBS (left) and Golomb code (right) system [32]. (**b**) Physical random code system [51].

It can be seen that the OSNR is inversely proportional to the length of FUT, and it is independent of the other parameters so that the OSNR constantly descends with an increase in the FUT. The OSNR can be improved using a low-noise coding sequence, such as a Golomb code [32], a physical random code [51], a dual-layer phase, and amplitude coding [52]. For example, a perfect Golomb code, with cyclic auto-correlation values of exactly zero for all off-peak delays, is employed in the PM BOCDA scheme. Although the off-peak interactions cannot be entirely eliminated, the OSNR may be improved by 3~4 times in Brillouin gain measurements. Figure 8a plots an example of the calculated intensity of an acoustic wave filed for a Golomb code and a PRBS of equal rates and periods. While both modulation schemes provide a stationary and localized CP at the fiber center, the off-peak stimulation (i.e., noise floor) is significantly suppressed when the Golomb code is used and the OSNR is improved by an order of magnitude of 20.5 [32]. Meanwhile, the true random codes, generated using an autonomous Boolean network oscillator as the physical entropy source, are employed to stimulate the acoustic wave and the intensity distribution, as shown in Figure 8b. At the intermediate position of the sensing fiber, the acoustic wave is steadily and permanently generated, and the noise structure at all non-correlation positions is largely inhibited [51]. 

#### 3.1.3. Chaos-Based BOCDA

For chaos-based BOCDA, the residual off-peak SBS amplification, i.e., the spurious peaks induced by the inherent TDS of the chaotic laser and irregular fluctuations resulting from its stochastic property, provides an additional noise mechanism, and the detrimental interactions persistently accumulate along the FUT. Consequently, the SBS gain at the CP position is highest and the gain section cannot be effectively distinguished only if the noise accumulation exceeds the gain signal. The optimal sensing distance is determined by the delay time of the external cavity and the effective sensing distance is largely limited by the TDS. In fact, the MR is limited to only 906 m under a delay time of 100 ns in the proof-of-concept experiment, where the optimal MR is 10 m theoretically. For the suppression of the background noise, a two-step approach is proposed in order to extend the sensing distance.

**Figure 9:**
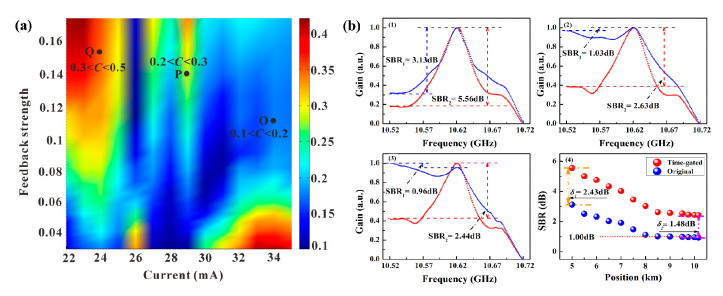
(**a**) The distribution map of the correlation coefficient under different injection currents and feedback strengths [33]. (**b**) The BGSs of the chaotic BOCDA systems with (red) and without (blue) the time-gated scheme at different fiber positions [34].

First, the amplitude of side-lobe peaks is directly proportional to the TDS value, which can be controlled by adjusting the injection current and feedback strength. The distribution of the correlation coefficient C at *τ_d_* = 115 ns, implying that the external cavity length is approximately 11.5 m, is illustrated in Figure 9a. The evolution of the TDS can be roughly divided into three zones, i.e., O, P, and Q. Among them, a TDS suppression region of 0.1 < *C* < 0.2, being the lowest in the single-feedback operating schemes, is employed as the laser source and the MR can be extended to 3.2 km with a SR of 7 cm [33]. 

Further, a time-gated scheme is proposed to restrict the residual off-peak noise floor, induced by an incompletely suppressed TDS and non-zero background [34]. The pump wave is modulated by an amplitude pulse, whose width is greater than the phonon lifetime. Owing to the square pulse switching, the cross-correlation between the pump and probe waves is confined to the pulse duration time, only operating at a high level and quickly decaying to zero at the terminal. Therefore, the residual SBS interaction is greatly suppressed, and a large enhancement of MR can be obtained. The modified experimental setup of the time-gated system is depicted in Figure 5 of [34], where the pulse duration is 120 ns for maximizing the valuable Brillouin gain signal. In the ordinary and time-gated schemes, the BGSs at 5.0, 8.5, and 10.0 km are measured and compared by the signal-to-background ratio (SBR), as shown in Figure 9(b1–b3). When the sensing fiber is longer than 8.0 km, the SBRs of the ordinary scheme are almost constant at 1.00 dB, implying that the gain signals can submerge into the noise floor. In contrast, the SBRs of the time-gated scheme are improved to 1.48 dB at the terminal of the FUT and the MR can approximately approach 10.2 km with a 9 cm SR [34].

Remarkably, the SR is only dependent on the chaos bandwidth and will not change with the sensing distance, theoretically. However, the incompletely suppressed noise background will continuously accumulate with the MR increase and result in SR deterioration. In future exploration, the residual TDS peaks and fluctuating noise floor may be further eliminated using an optimized time-gated scheme with a high extinction ratio and the differential denoising method, respectively. For chaos-based BOCDA systems, the core competitiveness suggests that the single CP over the arbitrarily long FUT is capable of coupling the large MR and the high SR. In addition, a longer sensing distance is simply achieved as a higher injection power is permitted under a high SBS threshold.

In this sub-section, several methods used for improving the measurement SNR are introduced. In sine-FM schemes, the noise background induced by the broadened non-local beat spectra is eliminated by differential measurements and a pure BGS can be obtained. Similarly, in PM and chaos-based schemes, the noise floor is inhibited by removing the amplitude fluctuations at off-peak positions. For sine-FM or PM schemes, although the BGS is optimized to a large extent and the SR easily reaches the centimeter level, the MR is also severely limited by the periodicity of the CP and the sensing distance is merely several hundred meters. Notably, due to the characteristic of a single CP over the whole FUT, the chaos-based schemes can achieve a large MR of more than 10 km only though restraining the noise background by TDS suppression and temporal gating. However, the utilization of PODG, which is applied to localize the single CP manually, is time-consuming and impractical.

### 3.2. Concurrent Interrogation of Multiple CPs

Although the noise floor is suppressed the furthest, the gain ambiguity between the adjacent CPs still seriously limits the MR in sine-FM and PM BOCDA systems. Creatively, a series of concurrent interrogation methods is proposed to separate and extract the gain signal from the multiple CPs in a single measurement. 

#### 3.2.1. Sine-FM BOCDA

**Figure 10:**
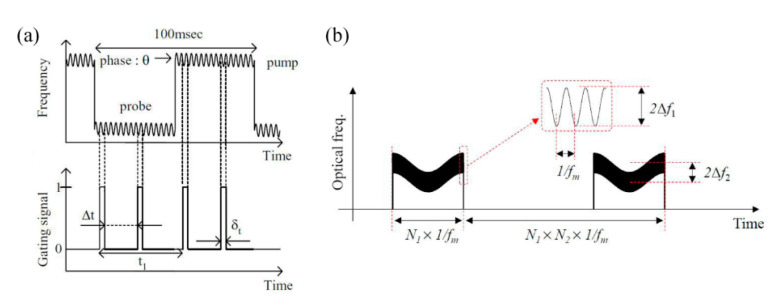
(**a**) Principle of temporal gating [21]. (**b**) Principle of the temporal gating based on double modulation [54].

In 2005, Prof. K. Hotate et al. proposed the time-gated sine-FM BOCDA schemes, where the amplitude pulse modulation was additionally overlaid on the pump waves [53]. As shown by the schematic diagram in Figure 10a, the sine-FM wave is modulated into the pulse, whose duration is equal to the period of sine-FM, and only one CP is selected among the *N* CPs to localize the sensing position, so that the MR may be promoted by *N* times. As a result, the MR is extended from 10 m to 250 m with a SR of 8 cm when $f_{m}$ is 10 MHz. In 2008, the pump and probe waves were both time-gated and the MR exceeded 1 km with a SR of 7 cm [21]. In 2015, an optimized differential measurement scheme based on temporal gating and double modulation was proposed to further enlarge the MR, as illustrated by Figure 10b [54]. In the double modulation scheme, two frequencies ($f_{m}$,$f_{m}/N_{1}$) are simultaneously overlaid to the laser source and the modulation amplitudes are $\Delta f_{1}$ and $\Delta f_{2}$, respectively [55]. The SR is determined only by the higher $f_{m}$, and the MR is extended by $N_{1}$ times, due to secondary modulation. One of the key constraints is that the Δz of slower modulation should be shorter than the $d_{m}$ of fast modulation to prevent the signals from inteferring with adjacent CPs. A time-gated scheme is also employed, and then the MR may be extended by $N_{1}\times N_{2}$ times. Finally, the distributed sensing with a 10.5 km MR is experimentally conducted and the MR is enlarged 2000 times, where the SR is less than 1 cm in the highest [54].

**Figure 11:**
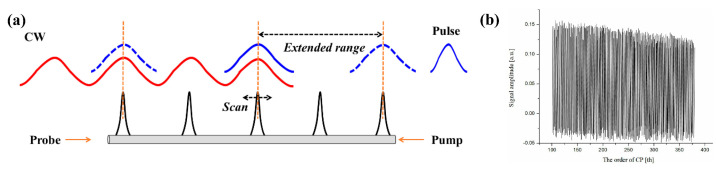
(**a**) Principle of the time-division multiplexing sine-FM BOCDA system. (**b**) The concatenation results of 148 BGSs along the whole FUT.

Based on the above researches, time-division multiplexing sine-FM BOCDA using time-domain data processing for the concurrent interrogation of multiple CPs is proposed and experimentally demonstrated [29]. As shown by the schematic diagram in Figure 11a, the pump wave is modulated by an amplitude pulse and the probe remains continuous. With transmitting the pump pulse, a series of CPs at different positions are sequentially generated and the Brillouin gain is resolved in the time trace of probe wave, which is acquired in a similar way to the BOTDA system. When the pulse width is shorter than $1/f_{m}$, each time section of the probe wave contains the Brillouin gain information of a single CP, so one can sequentially obtain the Brillouin gain of different CPs by analyzing the time trace of the probe wave. Compared with the time-gated scheme, the N CPs are simultaneously interrogated and the measurement speed is significantly improved, although the MR is also enlarged by N times. 

Figure 11b plots the concatenation results of 148 BGSs obtained from 148 CPs, where each BGS is obtained by analyzing multiple raw traces. It is observed that the gain signal gradually decreases as the order of CP increases, which can be attributed to the propagation loss of the pump pulse depletion. Because of this decrease in the Brillouin gain, the measurement error may increase in the long-range measurement, which limits the MR. The authors further achieved the optimal result that 948 CPs are simultaneously interrogated along the 10.15 km FUT with a SR of less than 5 cm [56]. It is noticeable that the SR of this proposed scheme is directly related to the duration of the pulse, which can cut off the optical spectrum or provide a non-uniform spectral coverage. Furthermore, the finite rising and falling edges of the pulse possibly induce a frequency chirp, which can reduce the Brillouin gain and distort the BGS [57].

As we know, in the BOCDA schemes of using time-domain data processing, the MR is practically restricted by two aspects. The pulse depletion of the pump wave results in a continuous attenuation of gain intensity; therefore, the SNR is severely deteriorated [41]. On the other hand, the peak power of the pump pulse is limited to ~20 dBm due to the detrimental effects of modulation instability [57]. In 2019, the distributed Raman amplification (DRA) was firstly introduced into sine-FM BOCDA scheme to achieve a 52.1 km MR, which is the longest sensing range demonstrated by BOCDA systems [35]. 

Owing to the transmission loss and the SBS interaction, the depletion of the pump pulse is gradually severe with an increase in the sensing distance. Interestingly, the frequency shift of Raman scattering is about 13 THz, which is significantly higher than BFS [58], and the stimulated Raman scattering (SRS) can enhance the Brillouin pump by selecting an appropriate pump source in the backward direction. It is noticeable that the SRS is stimulated along with the entire fiber, so the distributed Brillouin pump wave may be amplified, starting at the fiber end. 

**Figure 12:**
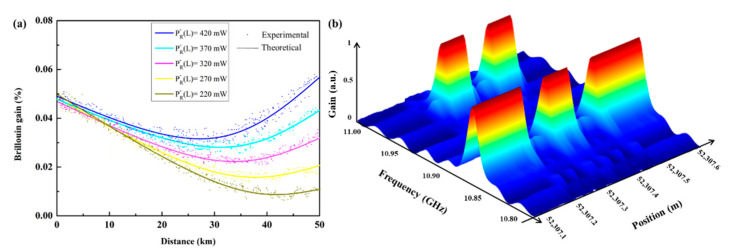
(**a**) The experimental and theoretical Brillouin gain trace along the whole FUT after DRA. (**b**) Measured BGS near the strain-applied section at the end of the FUT [35].

In the simulations and experiments, the Brillouin signal is extracted using the peak value of each BGS, and the gain trace along the whole FUT is illustrated in Figure 12a. As seen from the simulation results, the gain signal significantly ascends with an increase in the power of the Raman pump, and the gain intensity performs the lowest at the central positions. Due to the centimeter-level SR (narrow acoustic field), the intensity of the gain signal is feeble and fluctuates. Benefiting from the effective enhancement of SNR at the far end of the FUT, the strain-applied section can be successfully identified, as shown in Figure 12b. The FUT is 52.1 km and the BFS of the two 7 cm zones is completely separate from the normal section, implying that the SR is about 7 cm. Note that only the first-order backward Raman pump is applied to compensate for the Brillouin depletion of the pump pulse to extend the MR to 52.1 km [35].

In addition, a frequency division multiplexing scheme has been proposed to generate a sequence of CPs and distinguished by the difference in BFS, where the FUT consists of three fiber sections with distinguishable BFSs and corresponding dm lengths. Therefore, three BGSs of different BFS can be simultaneously obtained by matching the sweep frequency so that the MR is triple the size of the single-CP scheme. Similarly, a bidirectional detection scheme is proposed and doubles the MR by independently analyzing the Brillouin gain and the loss spectra of two adjacent CPs. However, the MR improvement in the above two schemes is extremely limited by the $d_{m}$ and the sensing distance remains at hundreds of meters.

#### 3.2.2. PM BOCDA

**Figure 13:**
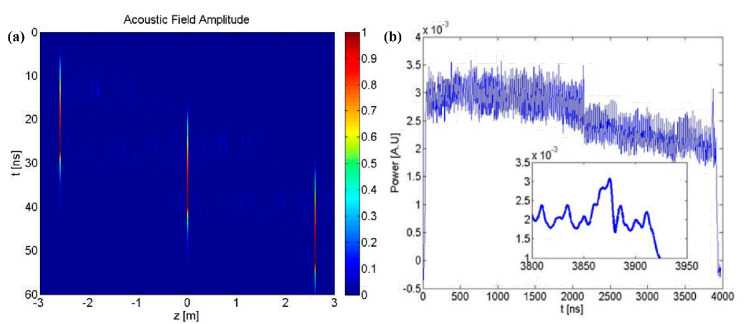
(**a**) Simulated magnitude of the acoustic wave as a function of position and time along a 6 m-long fiber section. (**b**) Measurements of the output signal power as a function of time [55].

Similarly, the time-division multiplexing PM BOCDA has also been proposed to extend the MR. Prior to this, a perfect Golomb code was employed in the phase modulation of both pump and probe to improve the OSNR, and then an amplitude pulse was superimposed on the phase-modulated pump wave. Figure 13a shows the magnitude of acoustic field over a 6 m-long fiber section [59]. As expected in the time-division multiplexing scheme, the acoustic field is confined to a discrete set of spatially periodic CPs. The separation between neighboring peaks is N⋅Δz. The pulse width is restricted to the order of the bit length, and the multiple CPs do not overlap in the time domain. Figure 13b plots the output signal power as a function of time. The FUT is 400 m and equally consists of two segments with a BFS of 10.90 GHz and 10.84 GHz, respectively, where a 5 cm heated section is placed at the end. It can be seen that the multiple CPs in the output probe power are sequentially distributed as the positions and each peak corresponds to the SBS interaction at a single CP. At the heated section, a specific gain peak appears and the BFS becomes more pronounced than all other locations. Note that each trace is averaged over 64 pump pulses in order to overcome the measurement noise, such as polarization scrambling variations, coding, and thermal noises. Finally, the BGS is experimentally acquired over a 400 m MR FUT with a 2 cm SR [59]. 

Additionally, in order to improve the SNR and measurement speed, a protocol of combining amplitude and phase sequence coding is further proposed [60]. When the noise floor induced by the off-peak Brillouin amplification is adequately restricted through the time-gated scheme and/or the Golomb code, the OSNR is typically limited by the additive noise sources associated with the measurement setup [61]. Similar to the time-gated scheme using a single pulse, the pump wave can be modulated by an aperiodic sequence on top of the PM waves, and then the CP may be sequentially generated, which is coincident with the propagation of the aperiodic pulse. Consequently, the acoustic field is stimulated and switched following the pattern of the incoherent pump pulse. The SBS gain at each CP is also resolved in the time trace of the probe wave and the measurement SNR can be further improved due to weaker residual side-lobes. Finally, a distributed measurement of 8.8 km FUT is obtained with a SR of 2 cm [61]. 

**Figure 14:**
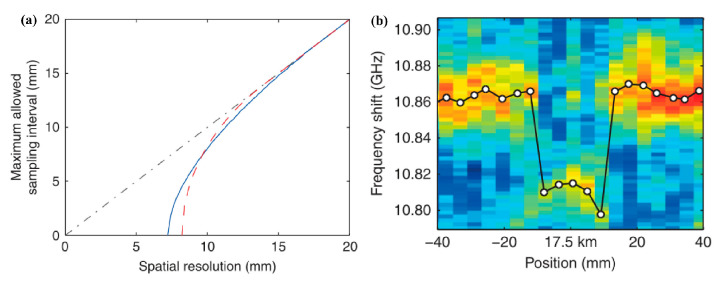
(**a**) Maximum allowed sampling interval required to obtain a given SR. (**b**) Measured BGS as a function of position and BFS [30].

Further, an optimal PM Brillouin sensor combining the time domain and correlation domain analysis has been proposed [30]. First, the relationship between the SR and sampling interval is revisited and the latter one is discreetly selected. As shown in Figure 14a, when a bit duration of 140 ps is adopted, the optimum maximum sampling interval is equal to the given SR. Remarkably, the asymptotic curve, plotted by a black dash-dotted line, performs the conditions commonly used in the current. The pulse duration is also optimized to achieve the best performance. For a shorter pulse, the acoustic wave at each CP position does not have enough time to reach the steady-state condition, leading to strong background noise and a reduction in the SNR. Then, based on the optimized parameters, such as the sampling interval, the pump pulse width, and the bit length of the PRBS, a distributed measurement over a 17.5 km fiber is conducted [30]. Figure 14b plots the map view of BGSs near the connector between the FUT and isolator, and the retrieved BFS at each localization is plotted by white circles. Clearly, a 16 mm-long section under the two mated connectors is distinguished by a very sharp variation of BFS. Finally, the optimized result of 8.3 mm SR over a 17.5 km MR is achieved [30]. 

In this sub-section, the time-division multiplexing methods are introduced in sine-FM and PM schemes, where the gain crosstalk between the multiple CPs is separated by time-domain data processing, concurrently interrogating multiple positions. Consequently, the MR is significantly extended to tens of kilometers, i.e., 52.1 km of the sine-FM scheme and 17.5 km of the PM scheme, with a centimeter-level SR, whose performance is notably better than that of SR-enhanced BOTDA schemes. With increasing the sensing length, the attenuation of the gain signal induced by the transmission loss and pump depletion will result in the continuous deterioration of SNR, and further improvements in the MR may be limited.

## 4. Discussions

In this paper, a brief overview of the developments of long-range BOCDA is provided, as summarized in Table 1.

(1) In the sine-FM BOCDA systems, the temporal gating scheme is incipiently proposed to extend the interval of adjacent CPs and then the double modulation scheme is overlaid to maximize the MR in the single-CP system, where the differential measurement is employed to eliminate the intrinsic noise structure and the measurement SNR is significantly promoted. Further, the time-division multiplexing scheme is demonstrated to simultaneously interrogate a sequence of CPs at a single time trace of probe power, supplementing the DRA to compensate for the pump loss; therefore, the MR extends to 52.1 km, which is the longest sensing range conducted by BOCDA systems.
(2) In the PM BOCDA systems, the OSNR is promoted by using the perfect Golomb code or incoherent pulse compression, i.e., a combination of amplitude and phase sequence coding. Based on the previous trials of time-domain data processing, an optimized time-division multiplexing scheme, including a careful choice of the sampling interval, pulse width, and PRBS length, experimentally enlarges the MR to 17.5 km with a SR of 8.3 mm, which is the best coupling performance between the MR and SR in Brillouin sensors.
(3) In the broadband source-based BOCDA systems, the noise-like property ensures that there is a sole CP along the fiber, so the MR can be extended due to the gain anti-interference in principle. ASE-based BOCDA is proposed and demonstrated with a 5 cm short-range precise measurement in the proof-of-concept experiment. Notably, the chaos-based BOCDA promotes forceful competitiveness in coupling the long MR and high SR, owing to the superior SNR. After suppressing the chaos TDS, a time-gated scheme is proposed to restrain the noise background and promote the MR to 10.2 km with a SR of 9 cm. In addition, the inherently high-incoherence source, used as the sensing signal, can largely avoid the modulation bandwidth limitation to simply achieve a millimeter-scale SR.

The main challenges and improvements are as follows:

(1) Inferior SNR. In all the BOCDA schemes, a narrow and feeble acoustic field is essential to achieve a centimeter-level SR so that a trade-off problem is severe between the SNR and sensing distance.
(2) Time-consuming measurement. The measurement speed of BOCDA schemes is limited by two aspects: one is the scanning of CP interval and the other is the frequency sweeping near the central BFS, although the inspired methods, including time-domain data processing using the single-pulse or double-pulse pair [62], as well as scanning-free BOCDA [63,64,65], have been verifiably proposed to shorten the measurement time.
(3) SR deterioration. In the sine-FM and PM systems, the scanning of periodic CPs is achieved by adjusting the frequency of the sine wave or the rate of the bit sequence. As a result, the CP width also changes slightly during the localization process, leading to the SR worsening in principle [29]. Consequently, the SR will be gradually deteriorated by increasing the sensing distance, which limits the further expansion of MR, despite the fact that the phase-shift keying [66] and short-pulse optical source [67] have been adopted to achieve a sub-millimeter SR. It is noticeable that the SR of the chaos-based scheme is only dependent on the chaos bandwidth and will not change with an increase in the MR [49], implying that the chaos-based BOCDA has performed forceful competitiveness in coupling the long MR and high SR.
(4) Ultra-long delay line. In the sine-FM and PM systems, in order to achieve a fully distributed measurement, the zeroth-order CP, invariably operating at the middle of the fiber, must be moved outside the FUT and only the higher-order CPs can ensure a lower measurement error in localization or SR. Consequently, the ultra-long delay fiber is obligatory to select the high-order CP in current schemes. For example, a 250 km delay fiber is opted in 50 km MR schemes [35]. In chaos-based BOCDA, the delay line, whose length may be shorter than the periodic schemes, must be programmable to localize the single CP by adjusting the delay length.
(5) To obtain a higher performance, the stability, simplicity, and cost should also be considered, which is equally important in practical applications.
(6) To open up more suitable applications. The unique advantage of ultra-high SR and distributed sensing can be employed for precise measurement, including simultaneous strain and temperature measurement [68], the distributed analysis of SBS over different waveguides [44,69,70,71], opto-mechanics community [72,73,74], and so on.

## 5. Conclusions

In this review, the basic principles of SBS interactions and correlation domain operating are introduced. Then, the several enhanced techniques underpinning MR enlargement, for example enhancing the interval of adjacent CPs, improving measurement SNR, and concurrent interrogating multiple CPs, are overviewed, respectively. Finally, the recent progresses and main merits or disadvantages in present BOCDA schemes are summarized. The avenues for future improvement are also prospected. In conclusion, the core competence of BOCDA is the incomparable convenience in achieving millimeter-level SR. Among the extensive applications, the BOCDA sensor can achieve distributed sensing with a long range of several tens of kilometers, as well as distinct temperature and strain states, which may offer broad prospects in practical applications, including the fire alarming, pipeline leakage detection, structure health monitoring, and so on.

## Figures and Tables

**Table 1:** Summary of the recent progresses and main merits or disadvantages in BOCDA.

Categories	Enhanced Techniques	Performances ^1^	Merits	Disadvantages	
Sine-FM BOCDA	Temporal gating	1 km@7 cm [21]	Longest MR	SR deterioration Bandwidth limitation	Inferior SNR Ultra-long delay line Time-consuming
	Differential measurement	10.5 km@1 cm [54]			
	Time-division multiplexing	10.15 km@5 cm [56]			
	Distributed Raman amplification	52.1 km@7 cm [35]			
PM BOCDA	Golomb codes	0.4 km@2 cm [59]	Best coupling of MR/SR		
	Incoherent pulse compression	8.8 km@2 cm [60]			
	Time-division multiplexing	17.5 km@0.83 cm [30]			
Chaos-based BOCDA	Ordinary chaos	0.9 km@4 cm [26]	Inherently high- incoherence source	Localization by variable delay line	
	TDS suppression	3.2 km@7 cm [33]			
	Temporal gating	10.2 km@9 cm [34]			

^1^ The performance of BOCDA is referred as MR@SR.

## Data Availability

Not applicable.
